# A Machine Learning Approach to Parkinson’s Disease Blood Transcriptomics

**DOI:** 10.3390/genes13050727

**Published:** 2022-04-21

**Authors:** Ester Pantaleo, Alfonso Monaco, Nicola Amoroso, Angela Lombardi, Loredana Bellantuono, Daniele Urso, Claudio Lo Giudice, Ernesto Picardi, Benedetta Tafuri, Salvatore Nigro, Graziano Pesole, Sabina Tangaro, Giancarlo Logroscino, Roberto Bellotti

**Affiliations:** 1 Istituto Nazionale di Fisica Nucleare (INFN), Sezione di Bari, Via A. Orabona 4, 70125 Bari, Italy; ester.pantaleo@uniba.it (E.P.); alfonso.monaco@ba.infn.it (A.M.); nicola.amoroso@uniba.it (N.A.); loredana.bellantuono@uniba.it (L.B.); sabina.tangaro@uniba.it (S.T.); roberto.bellotti@uniba.it (R.B.); 2 Dipartimento di Scienze Mediche di Base, Neuroscienze e Organi di Senso, Università degli Studi di Bari Aldo Moro, Piazza G. Cesare 11, 70124 Bari, Italy; giancarlo.logroscino@uniba.it; 3 Dipartimento Interateneo di Fisica M. Merlin, Università degli Studi di Bari Aldo Moro, Via G. Amendola 173, 70125 Bari, Italy; 4 Dipartimento di Farmacia-Scienze del Farmaco, Università degli Studi di Bari Aldo Moro, Via A. Orabona 4, 70125 Bari, Italy; 5 Centro per le Malattie Neurodegenerative e l’Invecchiamento Cerebrale, Dipartimento di Ricerca Clinica in Neurologia, Università degli Studi di Bari Aldo Moro, Pia Fondazione Cardinale G. Panico, 73039 Tricase, Italy; daniele.urso@kcl.ac.uk (D.U.); benedetta.tafuri@gmail.com (B.T.); salvatoreangelo.nigro@gmail.com (S.N.); 6 Institute of Psychiatry, Psychology and Neuroscience, King’s College London, De Crespigny Park, London SE5 8AF, UK; 7 Dipartimento di Bioscienze, Biotecnologie e Biofarmaceutica, Università degli Studi di Bari Aldo Moro, Via A. Orabona 4, 70125 Bari, Italy; claudio.logiudice@uniba.it (C.L.G.); ernesto.picardi@uniba.it (E.P.); graziano.pesole@uniba.it (G.P.); 8 Istituto di Biomembrane, Bioenergetica e Biotecnologie Molecolari, Consiglio Nazionale delle Ricerche, Via G. Amendola 122/O, 70126 Bari, Italy; 9 Istituto di Nanotecnologia (NANOTEC), Consiglio Nazionale delle Ricerche, Via Monteroni, 73100 Lecce, Italy; 10 Dipartimento di Scienze del Suolo, della Pianta e degli Alimenti, Università degli Studi di Bari Aldo Moro, Via A. Orabona 4, 70125 Bari, Italy

**Keywords:** blood transcriptomics, Parkinson’s disease, machine learning, xgboost, feature selection, oxidative stress, inflammation, mitochondrial dysfunction

## Abstract

The increased incidence and the significant health burden associated with Parkinson’s disease (PD) have stimulated substantial research efforts towards the identification of effective treatments and diagnostic procedures. Despite technological advancements, a cure is still not available and PD is often diagnosed a long time after onset when irreversible damage has already occurred. Blood transcriptomics represents a potentially disruptive technology for the early diagnosis of PD. We used transcriptome data from the PPMI study, a large cohort study with early PD subjects and age matched controls (HC), to perform the classification of PD vs. HC in around 550 samples. Using a nested feature selection procedure based on Random Forests and XGBoost we reached an AUC of 72% and found 493 candidate genes. We further discussed the importance of the selected genes through a functional analysis based on GOs and KEGG pathways.

## 1. Introduction

Parkinson’s disease (PD) is a chronic, degenerative disease of the central nervous system with a pattern of incidence that increases with age; as the population ages, its burden is poised to increase [1]. Despite considerable research efforts, PD is incurable; available treatments can only help manage the symptoms, and its diagnosis often occurs a long time after onset after substantial loss of function of substantia nigra dopamine neurons [2].

Massively parallel analysis of cellular RNAs can provide an unbiased set of biomarkers of PD and can generate hypotheses about disease mechanisms. It may be particularly useful for decoding a disease with considerable environmental and epigenetic contributions not readily explained by variations in the genomic fingerprint such as PD [3]. Brain transcriptomics has already shown its potential to uncover the functional mechanisms at the basis of this disease although its signal is confounded by underlying differences in cell type composition and it can only be performed after death [4]. Whole blood transcriptomics represents a convenient and less invasive alternative to brain transcriptomics for early PD diagnosis, as blood is a readily accessible peripheral biofluid and blood and brain share significant transcriptional profile similarities [5,6] although more investigations are needed in this field. A number of experimental observations have shown molecular and biochemical changes in the blood cells of PD subjects [7,8] and RNA-sequencing experiments on blood leukocytes have revealed the diagnostic potential of long non coding RNAs (lncRNAs) [9]. Some studies have identified biomarkers from blood that are robust and have great potential for helping reduce misdiagnosis [10,11,12].

As high throughput technologies such as transcriptome sequencing can now generate huge amounts of biological data at relatively low costs, the processing and extraction of relevant signal requires the adoption of artificial intelligence methodologies. A number of Machine Learning (ML) approaches have been undertaken for PD classification that use as input vocal and gait [13] or neuroimaging [14] features, or genetic risk scores from Genome Wide Association Studies (GWAS) studies [15] and microarray transcriptional profiles [16,17]. We used advanced Machine Learning techniques for feature selection and classification of early (drug-naive) PD patients and healthy controls (HCs) using gene expression data from blood RNA sequencing.

For blood transcriptomics, experience suggests that large cohorts are needed, and that drug-naive patients should be used, as medications certainly affect gene expression [18]. Microarray assays for whole blood transcriptomics have been used to classify early stage drug-naive PDs vs. HCs [19,20] with a small number of samples (less than 50 PD subjects), while previous experiments with a large number of samples used PD subjects on dopaminergic medication [17].

Given the importance of using large cohorts of drug-naive patients, we used open access gene expression data from the Parkinson’s Progression Markers Initiative (PPMI), an international study that has enrolled the largest to date cohort of untreated PD patients (around 430 subjects) across multiple sites (www.ppmi-info.org/data accessed on 11 March 2022) [21].

## 2. Materials and Methods

### 2.1. PPMI Data

We downloaded PPMI whole blood transcriptome data from the LONI Image and Data Archive (IDA) (data dowloaded in July 2021). From the available set of sequenced samples, we selected 579 samples collected from different individuals, namely 390 subjects in the early PD cohort and 189 age-matched subjects in the HC cohort. Therefore the dataset consisted of twice as many PD cases as HCs. Each sample had expression values (read counts) for a total of around 60,000 transcripts. The early PD cohort included subjects with PD that were not treated with dopaminergic medications, that were not carriers of ‘LRRK2’, ‘GBA’ or ‘SNCA’ mutations, and that did not have a first relative with one or more mutations. Sequence data had been aligned to GRCh37(hs37d5) by STAR (v2.4K) [22] using exon-exon junctions from GENCODE v19 and gene count data had been obtained via featureCounts [23] by the same GENCODE annotations. Samples that failed quality control were excluded [24].

Subject metadata that we downloaded from the PPMI website included biological variables such as age, sex, clinical site and clinical measures of motor symptoms such as indicators of tremor dominant (TD) or postural instability gait difficulty (PIGD), of non motor symptoms such as categorical REM sleep behavior disorder (RBD), of cognitive impairment (CI) such as the Montreal Cognitive Assessment or MoCA index (adjusted for education), and of olfactory function (UPSIT or University of Pennsylvania Smell Identification Test score). Additional metadata included technical variables such as, for instance, RIN (RNA integrity number), percent usable bases, total number of reads, sequencing plate. Table 1 reports some statistics on the metadata.

For up-to-date information on the study and for access to the data, visit www.ppmi-info.org accessed on 11 March 2022.

### 2.2. Overview of the Methodology

**Figure 1:**
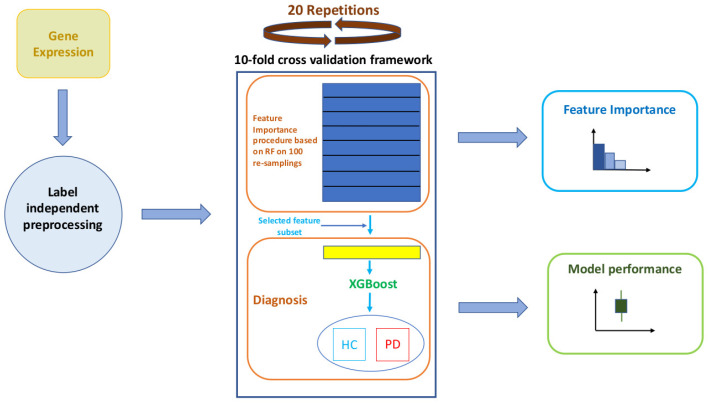
Schematic workflow of the performed analyses. The main phases are: (i) preprocessing, (ii) learning and (iii) performance evaluation.

Our computational workflow consists of three main phases: (i) a first preprocessing phase, which was essential to manage the informative content of highly heterogeneous and computationally demanding data such as transcriptomes; (ii) a second learning phase, which exploited a feature importance evaluation embedded in a Random Forest (RF) classification procedure [25,26] and whose best features were used to feed an eXtreme Gradient Boosting (XGBoost) algorithm [27]; (iii) finally, an unbiased evaluation of classification performances and of the set of important features obtained through a nested cross-validation scheme. A schematic overview of our workflow is presented in Figure 1. A detailed description of the previously mentioned processing steps is presented in the following methodological subsections.

For all analyses we used R version 4.0.3, packages xgboost v1.6.0.1, caret v6.0-90, and Bioconductor packages DESeq2 v1.30.1, limma v4.46.0, enrichR v3.0, AnnotationDbi v1.52.0, and org.Hs.eg.db v3.12.0. The code used to conduct this research is available upon request.

### 2.3. Empowering Informative Content of Gene Expression Values

The first phase of our workflow consists of multiple preprocessing procedures. This phase is essential given the large number of features, namely gene expression values based on the GENCODE v19 comprehensive annotation. A number of label independent filtering steps, where the labels are “PD” and “HC”, were required to extract informative content.

First, we selected only transcripts corresponding to protein coding genes and long intergenic non-coding RNAs (lincRNAs). Second, we discarded 2667 transcripts driving technical variance [24], which left us with 18,727 protein coding genes and 7444 lincRNAs. Third, we removed lowly expressed genes, by keeping only genes that had more than five counts in at least 10% of the individuals, which left us with 21,273 genes. Fourth, we estimated size factors, normalized the library size bias using these factors, performed independent filtering to remove lowly expressed genes using the mean of normalized counts as a filter statistic. This left us with 12,612 genes. Finally, we applied a variance stabilizing transformation to accommodate the problem of unequal variance across the range of mean values. We used DESeq2 to perform theses steps [28].

**Figure 2:**
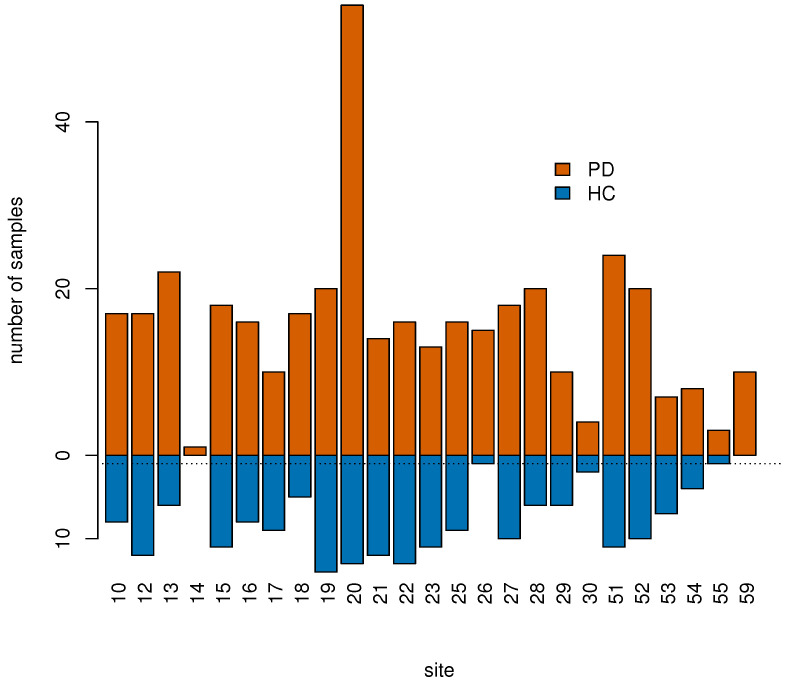
Samples were collected across 25 different sites labeled with an integer number. Sites “14”, “26”, “55”, and “59” had 0 or 1 control sample only (horizontal dotted line) and were excluded from the classification analysis as batch effects due to site could not be estimated and therefore corrected for.

Afterwards, we used control samples to estimate the batch effect of the site, that we subsequently removed in both controls and cases [29] using limma [30]. To perform this step we removed subjects from sites with no control samples or with only one control sample, i.e., sites “14” (1 sample), “26” (16 samples), “55” (4 samples), and “59” (10 samples), see Figure 2.

After this step, we were left with a total of 548 samples. Then we removed further confounding effects due to sex and RIN value, again with limma. Thus, for the subsequent analyses we considered a database including 548 subjects described by 12,612 genes.

### 2.4. Differential Expression Analysis

Before moving to the second phase of our workflow, namely the learning phase (see next section), we performed differential expression (DE) analysis, which is a classical and univariate approach towards the identification of biomarkers from RNASeq data. We will also test the performance of our ML approach (XGBoost) when we used as input the set of DE genes obtained with DESeq2 instead of the set of genes selected with RF. In the discussion we will contrast the results of this univariate approach with results from our machine learning multivariate approach. For DE analysis we used DESeq2 [28], a popular tool. As it is standard procedure, we used as input to the algorithm counts prior to independent filtering, batch correction and variance stabilization and defined a design matrix with four variables: the normalized RIN value, factor site, factor gender and the disease label. For the comparison between PD and HC, DESeq2 returns a positive fold change value to indicate an increase of expression of a gene in PD subjects vs. HC, and a negative fold change to indicate a decrease in expression. It also uses a shrinkage procedure to combine information from multiple genes, but its approach is univariate as it tests each gene individually for DE using a beta binomial generalized linear model. DESeq2 corrects for multiple testing using a Benjamini–Hochberg adjusted *p*-value. Genes with adjusted *p*-value < 0.05 are called significantly differentially expressed in the two classes. We will evaluate the fold change of genes with its associated error and adjusted *p*-value and compare results with a multivariate analysis that uses Machine Learning algorithms.

### 2.5. A Robust Learning Scheme

After performing DE analysis, we moved to the second phase of our workflow, the learning phase.

Our filtering procedure described in Section 2.3 had already significantly reduced the amount of gene expression to consider. Nonetheless, we designed and implemented an additional feature selection procedure (nested within the learning phase) to further reduce the number of genes with the two-fold goal of enhancing classification performances and optimizing model interpretability.

Within a repeated stratified (to tackle the control-patient mismatch) 10-fold cross-validation framework (20 iterations), we trained multiple RF models (100 repetitions, where each repetition used a different seed of the random generation process) to evaluate permutation feature importance measures. We chose RF for two main reasons: on the one hand, RF is easy to tune as it only depends on two parameters, namely the number of trees to grow and the number of features randomly selected at each split; on the other hand, RF is an extremely efficient algorithm on high dimensional data. Each forest was grown using 1000 trees, a sufficient value to allow the algorithm to reach a stable plateau of the out-of-bag internal error. The features selected at each split were $\sqrt{f}$ with *f* being the overall number of genes, which is the default value for this parameter. As already mentioned, another important advantage of the RF classifier is its embedded feature importance evaluation; during the training phase, the algorithm can assess how much each feature decreases the impurity of a tree, or the likelihood of incorrect classification of a new instance of a random variable and then can make an average over all trees [26]. Using this embedded feature importance procedure, we determined the overall feature importance ranking by averaging over the 100 repetitions. Then, a subset of size *C* of the most important features was used to train an XGBoost model; the XGBoost classification performance was evaluated on the validation set, for the twenty 10-fold cross validation iterations, in order to obtain an unbiased performance evaluation. As with RF, the XGBoost algorithm belongs to the set of learning approaches called *ensemble,* which combines and manages the predictions of several weak models to obtain a more robust model. While RF relies on bagging (Bootstrap aggregation), XGBoost exploits the Gradient Boosting framework. In the Gradient Boosting method, new models are applied to predicting residuals or errors of previous models and then added together to obtain the final predictive model. This approach implements a gradient descent algorithm to minimize the loss when including new models [27].

Overall, our procedure is very robust because, in addition to the high number of iterations implemented, we also use two different classification algorithms in the training and test phases which makes the results independent from the model. Then, to compare the performance of the ML approach to the performance of a simpler XGBoost classification algorithm that uses as input features the set of DE genes obtained with DESeq2, we trained the algorithm on 90% of the data and tested it on 10%.

Finally, we tested if the predicted probability of the algorithm was different between PD subjects with different endo-phenotypes: (i) MoCA ≤ 26 and MoCA higher than 26; (ii) PDs with RBD an PDs without RBD; (iii) PDs with TD and PDs with PIGD or undetermined; (iv) PDs with Normosmia and PDs with Hyposmia or Anosmia; (v) PD subjects belonging to different age categories, namely age ≥ 56 and age < 56.

### 2.6. Performance Evaluation

The last phase of our workflow is performance evaluation. A binary classification problem has only two class labels; therefore, the resulting model decisions can fall into four categories: true positives (TP) when the model correctly predicts the positive class, erroneous positive predictions (false positives, FP) and, analogously, true negatives (TN) and false negatives (FN).

Given these four cases, one can define several metrics; in particular, we considered here [31]:

- Accuracy
  $$\frac{TP+TN}{TP+TN+FP+FN};$$
- Sensitivity
  $$\frac{TP}{TP+FN};$$
- Specificity
  $$\frac{TN}{TN+FP};$$
- Balanced Accuracy
  $$\frac{Sensitivity+Specificity}{2};$$
- F1
  $$\frac{2TP}{2TP+FP+FN};$$
- Area Under the Receiver Operating Characteristics (ROC) Curve (AUC), which plots sensitivity against specificity by varying the decision threshold.

Sensitivity and specificity evaluate how well the model performs on the positive and the negative class, respectively. The other metrics provide an overall performance evaluation. Although these “overall” metrics are roughly equivalent, their values can ease the comparison of our results with the state-of-the-art.

## 3. Results

### 3.1. Evaluating the Informative Content of Transcriptomic Data

The first research question addressed by this work concerned the evaluation of the informative content provided by blood transcriptomic data. We first assessed the informative content through a univariate DE analysis and we found a total of 1368 up-regulated genes and 911 down-regulated genes with an adjusted *p*-value less than 0.05. Of the DE genes, however, only one gene, namely ‘RAP1GAP’, had a log fold change (lfc) higher than 0.5 in absolute value (lfc = −0.65 ± 0.15, adjusted *p*-value∼$10^{-5}$). In general, the DE signal, except for this gene, was very low.

**Figure 3:**
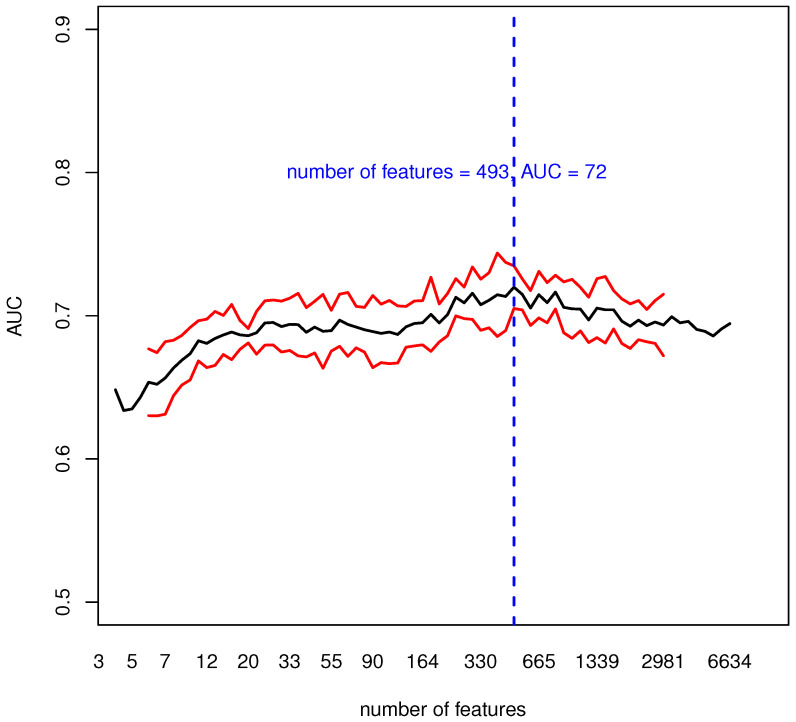
In black, the median AUC over 20 runs of 10-fold cross validation; in red, the median AUC ± its mean absolute deviation; in blue, the number of features (genes) where the maximum median AUC (72%) was reached. For each run, we collected the AUC values obtained at different thresholds *C* (or equivalently a different number of genes) and we interpolated these values to build a curve. Then we obtained the black curve as the median of 20 curves, one for each 10-fold Cross-Validation (CV) run.

We then evaluated the informative content of blood transcriptomic data using a multivariate ML procedure and the classification AUC as a performance measure, see Figure 3.

Figure 3 shows the cross-validation median AUC with its mean absolute deviation for a different number *C* of input features. The maximum median AUC of 72% with a mean absolute deviation of 1.5% is reached with a number of input features equal to *C* = 493. Despite classification results should depend on the number of features (genes) used to learn the model, this analysis shows that over an extremely broad range of features the informative content remains stable and accurate. For what concerns the other classification metrics obtained using the previously mentioned 493 features, a detailed overview is presented in Table 2.

The model is generally accurate, as shown by “global” metrics (AUC, F1, accuracy); it is worth noting the performance drop revealed by the balanced accuracy, which reflects the data imbalance. The same consideration holds for the performance gap in terms of sensitivity and specificity.

We tested the performance of an XGBoost classification algorithm that used as input features the set of DE genes obtained with DESeq2. We obtained an AUC of 64%, which is considerably lower than the performance of our ML approach based on RF and XGBoost, which proves how a multivariate ML model can be more effective on this type of data compared to classical DE approaches.

A final note on the performance of the algorithm with respect to PD endo-phenotypes. The predicted probability of the algorithm was higher for PD subjects in different age categories: the algorithm had an average predicted probability higher for PD individuals with age ≥ 56 (*p*-value 0.004, Wilcoxon test, average predicted probability = 0.77 for age < 56 and 0.84 for age ≥ 56), while there was no significant difference between PD subjects belonging to the other considered endo-phenotypic classes.

### 3.2. Evaluating Gene Importance

**Figure 4:**
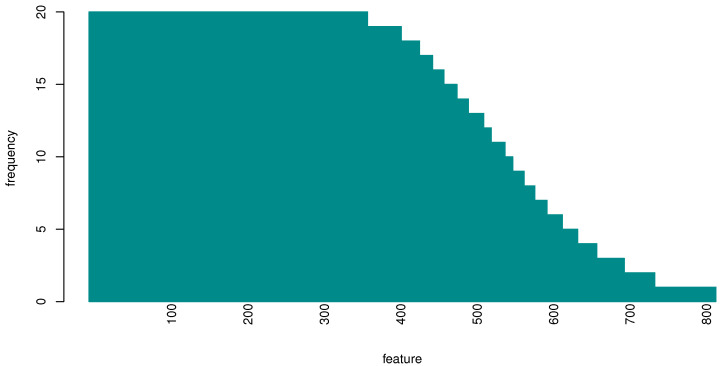
Histogram of the frequency of occurrence of the top 493 genes over 20 repetitions. At each repetition we collected the 493 most important genes; over 20 repetitions we gathered in total around 800 genes, many of which (365) appeared in all 20 repetitions.

As the RF feature importance procedure in principle returns a different feature ranking at each iteration (both because of the different cross-validation splits and intrinsic RF variability), we designed an experiment to investigate which were the most important genes for classification. Provided that the highest performance value was obtained with 493 features, within the cross-validation scheme, we evaluated the probability that an input feature (gene) is one of the top 493 genes, see Figure 4.

Among the most frequently selected genes, the 20 most important genes (according to the average importance ranking) are listed in Table 3; a list with the genes that have been selected in at least 70% of the iterations is presented in Table A1. This list includes 434 protein coding genes and 61 are lincRNAs (lincRNAs are marked with an asterisk).

### 3.3. Gene Set Enrichment Analysis

**Figure 5:**
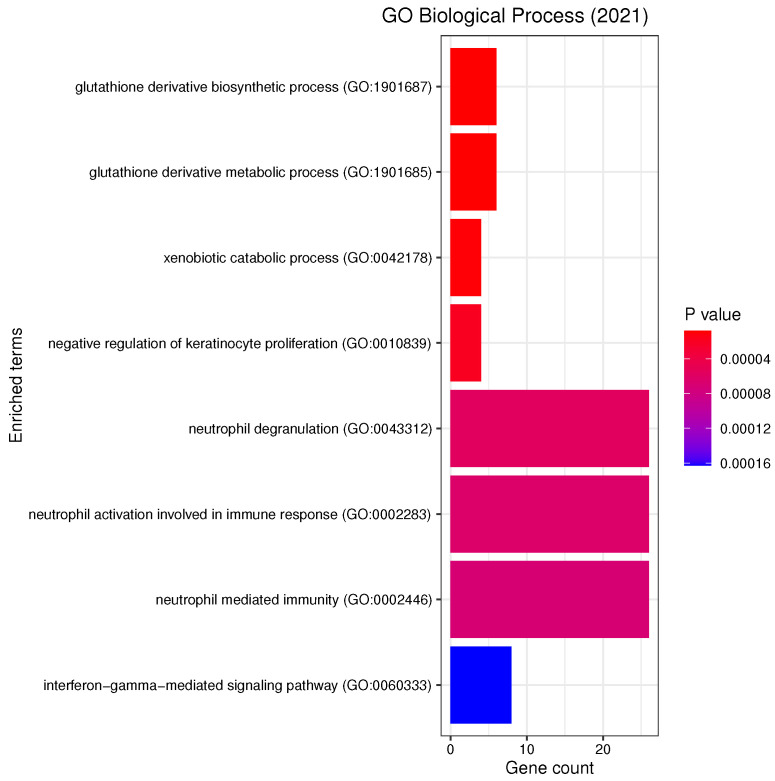
List of all the GO Biological Processes that are enriched in the selected genes, with the respective number of genes belonging to each term. The analysis was performed with enrichR at an FDR < 0.05.

**Figure 6:**
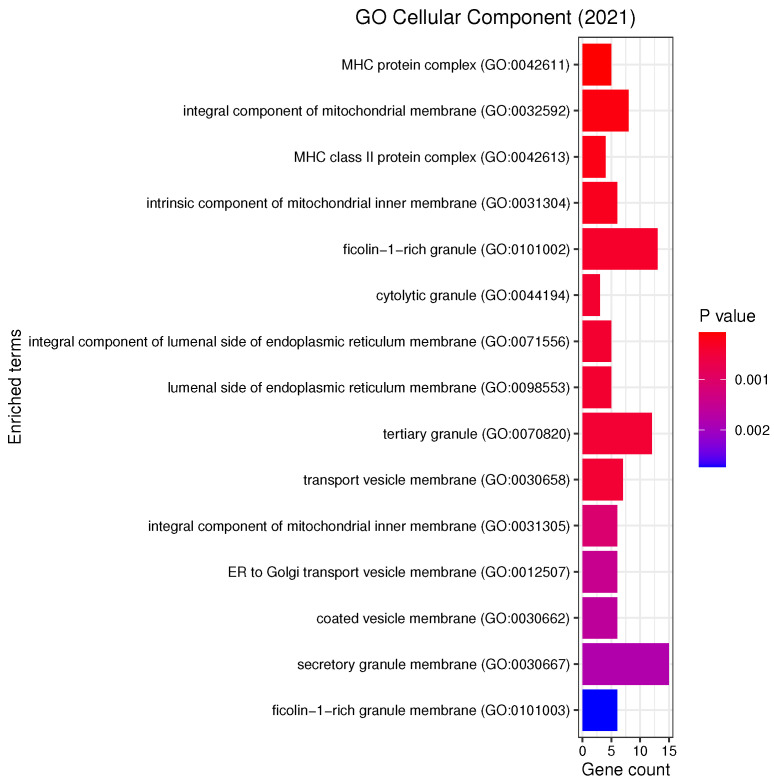
List of all the GO Cellular Components that are enriched in the selected genes with the respective number of genes belonging to each term. The analysis was performed with enrichR at an FDR < 0.05.

**Figure 7:**
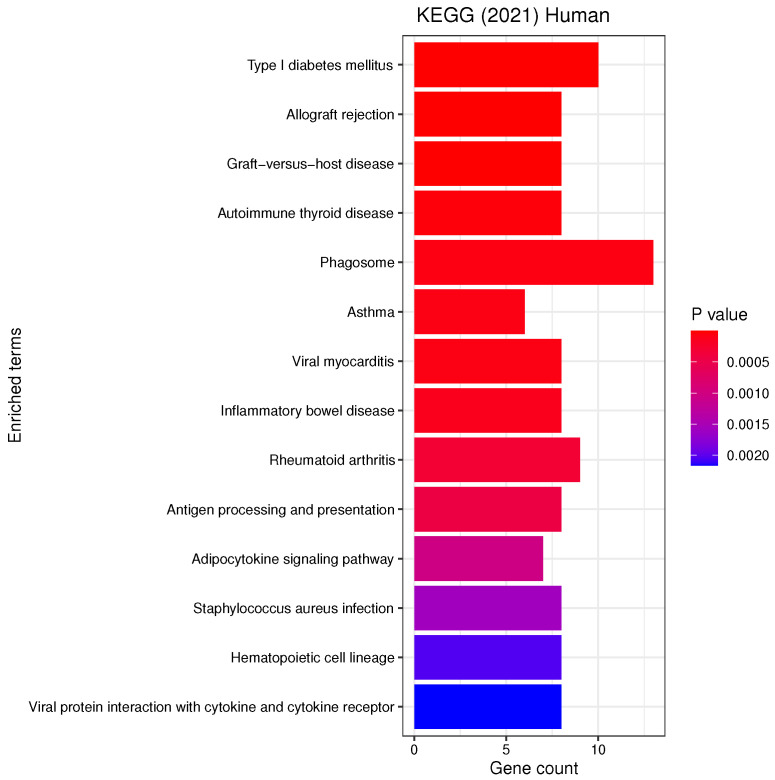
List of all the KEGG pathways that are enriched in the selected genes with the respective number of genes belonging to each term. The analysis was performed with enrichR at an FDR < 0.05.

We performed KEGG (Kyoto Encyclopedia of Genes and Genomes) pathway and GO (Gene Ontology) functional annotation enrichment analysis with respect to biological processes, cellular components and molecular functions using enrichR [32] on the list of most frequent genes (Table A1). Figure 5, Figure 6 and Figure 7 report all the resulting significant groups at a False Discovery Rate (FDR) < 0.05; no GO molecular function was significant.

## 4. Discussion

### 4.1. A Robust Machine Learning Model

With the robust methodology we implemented, we identified a set of around 500 genes that could discriminate between PD and HC with an AUC of 72%. Over 20 runs of cross validation (Figure 3) the AUC had a slightly increasing pattern for increasing values of *C*, and reached a maximum at a number of features *C* = 493, then slowly decreased. This behavior showed that the informative content of the selected genes was stable and accurate. While there was an imbalance between sensitivity and specificity, it was moderate and, if needed, this discrepancy could be mitigated with additional under-sampling of over-sampling techniques that could be embedded in the described methodology.

Comparing our performance with the state-of-the-art is not straightforward because of the nature of the data and because of ongoing research in the area. A comparable study on a large cohort of 523 individuals performed on blood microarray gene expression data and using Support Vector Machines reports an AUC of 79% on the validation set and of 74% on the test set [17]. However in that study PD subjects with a positive family history were not excluded and most importantly PD patients were treated with dopaminergic medication. Dopaminergic medication alters gene expression and thus confounds the underlying signal: higher discriminative performances are to be expected but are misleading.

A multivariate study not yet published [33] and performed on the same PPMI cohort as ours used a multi-modal approach that combines the informative content of transcriptomics, clinico-demographic data, genome sequencing data, and poligenic risk scores (PRS). To compare our results with theirs, we considered their transcriptomics-only model. They used Support Vector Machines (but they tested and tuned 12 different ML algorithms) and divided the PPMI cohort at baseline into a training (70%) and a validation set (30%), then they tested the resulting model on independent data from the PDBP (Parkinson’s Disease Biomarkers Program) cohort, although performances of the uni-modal model were not reported for this test set. After careful preprocessing, where they used limma to adjust for additional covariates of sex, plate, age, ten principal components, and percentage usable bases and then normalized counts, they used significantly over- or under-expressed protein coding genes as determined through logistic regression (*p*-value < 0.01) on the training set as input features to a Support Vector Machine classification algorithm. Using only transcriptome data they reached an AUC of 79.73% on the validation set, 73.89% accuracy, 54.60% balanced accuracy, 97% sensitivity, and 12% specificity. When they combined transcriptomics with the other multi-modal data using a union of the features as input features; after tuning, they reached an AUC of 85.03%, 75% accuracy, 68.09% balanced accuracy, 93% sensitivity and 43% specificity on the independent test set and determined, by comparing the relative importances of the input features, that the UPSIT score, as well as PRS, contributed most to the predictive power of the model, but the accuracy of these were supplemented by many smaller effect transcripts and risk SNPs.

The strength of our work is its high balanced accuracy in delineating cases and controls and its robustness. Our feature selection procedure identified a robust set of around 500 genes listed in Table A1 that may have some impact on PD biology.

### 4.2. Candidate Genes, GOs and KEGG Pathways

Accurate characterization of the selected genes and of significantly enriched gene sets is beyond the scope of this paper; however, we report a few comments on the enrichment analysis and a few notes on the selected genes.

Our analyses revealed a number of significant functions and pathways, some of which have already been linked to the pathogenesis of PD, such as oxidative stress, inflammation, mitochondrial and vesicular dysfunction, as well as associations between PD and diseases such as diabetes mellitus or inflammatory bowel disease (IBD) (see Figure 5, Figure 6 and Figure 7). *Oxidative stress* plays an important role in the degeneration of dopaminergic neurons [34]; its involvement in PD is further substantiated by Reactive Oxygen Species (ROS) induced Parkinsonian models and elevated oxidative markers in clinical PD samples [35]. Glutathione (GSH) is a ubiquitous thiol tripeptide that protects against oxidative stress-induced damage by neutralizing reactive oxygen species; its deficiency has been identified as an early event in the progression of PD [36]. *Inflammation* is another important contributor to the pathogenesis of the disease [37]. Interestingly, our GO analysis has identified biological processes that involve neutrophils. A very recent meta-analysis studying the association between the neutrophil-to-lymphocyte ratio (NLR), a well-established indicator of the overall inflammatory status of the organism, and clinical characteristics in PD has demonstrated that PD patients have an altered peripheral immune profile [38]. Neuronal expression of major histocompatibility complex I (MHC-I) and II (MHC-II) also play a neuroinflammatory role in PD [39,40]. The MHC gene family encodes molecules on the surface of cells that enable the immune system to recognize presented self- and foreign-derived peptides. MHC class II-positive microglia are a sensitive index of neuropathological change and are actively associated with damaged neurons and neurites in PD [41]. *Mitochondrial dysfunction* is another pathway that has been implicated in the pathophysiology of PD through both environmental exposure and genetic factors. The discovery of the role of the PD familial genes ’PTEN’-induced putative kinase 1 (PINK1) and parkin (PRKN) in mediating mitochondrial degradation reaffirmed the importance of this process in PD aetiology [42]. *Vesicular dysfunction* is another known contributor of PD [43]. Finally, *diabetes mellitus* and *inflammatory bowel diseases* (IBD) are known PD risk factors. In fact, population-based cohort studies indicate that diabetes and IBD are associated with increased PD risk by about 38% [44] and 22% [45], respectively.

A few notes on the set of genes selected follow. In Table 3 we reported the first 20 most important protein coding genes and lincRNAs in our analysis. We included lincRNAs because long non coding RNAs in general assume various roles, which include regulatory roles, and can thus modulate gene expression of protein coding genes; also they are very relevant in neurobiology, as many are associated with neurological pathologies [9].

‘MYOM1’, Myomesin1, the most important gene, is a protein coding gene and is up-regulated in PD subjects. Noticeably, ‘ENSG00000272688’ (Lnc-MYOM1-4) falls within an intron of MYOM1 and is the fifth most important lincRNA; gene ‘MYOM2’ is also in the list of selected genes and was selected in all the 20 repeated runs. Gene ‘MYOM1’ is significantly up-regulated in human substantia nigra pars compacta from PD patients [46] and is also one of the most important genes in [33], together with ‘SQLE’, ‘LGALS2’, and ‘NCR1’. The intersection between our and their set might be larger as in that paper only 29 out of a much larger set of genes selected are reported. Gene ‘SLC25A20’, Solute Carrier Family 25 Member 20, the second most important gene, was up-regulated in PD, and was one of the nine PD biomarkers identified by Jiang et al. [47], which used a meta-analysis of microarray gene expression data from [17,48,49]. ‘PTGDS’, another gene in our set, was also one of these nine biomarkers. In our set of genes, 6 other genes, ‘SLC18B1’, ‘SLC25A3’, ‘SLC11A2’, ‘SLC25A25’, ‘SLC25A43’, ‘SLC38A11’ belong to the solute carrier (SLC) superfamily, one of the major sub-groups of membrane proteins in mammalian cells. Their role in neurodegenerative disorders is described thoroughly in [50]. ‘NRM’, the third most important gene, the integral nuclear membrane protein Nurim, plays a role in the suppression of apoptosis [51], and apoptosis is the main mechanism of neuronal loss in Parkinson’s disease [52]. ‘PHF7’, PHD Finger Protein 7, is a candidate gene for a PD risk locus identified with a meta-analysis of genome-wide association studies [53]. Both protein coding gene ‘NUP50’ (Nucleoporin 50) and lincRNA ‘NUP50-DT’ (‘NUP50’ divergent transcript) are in our gene list. ‘CERS4’, Ceramide Synthase 4, is involved in Sphingolipid metabolism and its relation to PD is described in [54]. Dysregulation of metabolic pathways by carnitine palmitoyl-transferase 1 ‘CPT1A’ plays a key role in central nervous system disorders [55].

Gene ‘RAP1GAP’ has been identified by both the DE analysis and the ML methodology (it is selected 20 times over 20 repetitions) (see Table A1). This gene is under-expressed in PD subjects and has a role in orchestrating the development and maintenance of different populations of central and peripheral neurons [56].

### 4.3. Final Considerations

Two final comments. First, the performance of a classification algorithm that used as input features DE genes, as found by DESeq2, showed much lower performances compared to those obtained with the set of features selected with the ML algorithm, thus confirming the validity of our methodology and the importance of using ML models with gene expression data from RNA sequencing of whole blood where the signal is significantly low. Furthermore, we notice how some of the genes selected by the ML algorithm are not DE between the class of PD and the class of HC subjects (see Table A1) but nonetheless contain a relevant signal.

Last, the different average predicted probability between subjects that falls in different *age of onset* classes (early-onset and late-onset PD subtypes) could reflect the heterogeneity of PD at different ages. In fact, it has been observed that PD patients with older age onset have more severe motor and non-motor burdens and a more widespread involvement of striatal structures [57].

## 5. Conclusions

We used a robust ML approach to make predictions on PD from whole blood expression data. The studied cohort included 390 early stage drug-naive PD subjects and 189 age-matched HCs. After careful preprocessing, including batch correction and independent gene filtering, we used a feature selection procedure based on RF and re-sampling and an XGBoost algorithm to evaluate PD vs. HC classification performances within a nested 10-fold cross validation scheme. We explored classification performances for different values of *C*, the number of features selected, and identified a set of around 500 genes listed in Table A1 that corresponded to maximum discriminative power. We also performed an enrichment analysis on this set of genes and identified significant GO terms and KEGG pathways, many of which are in line with the current literature on PD, although further analysis of these sets is needed and is outside the scope of our work. A strength of our methodology is its robustness. The balanced accuracy of our algorithm compares favorably with the state-of-the-art.

This area of research is cutting edge and requires further investigation. A possible extension of our work could be the evaluation of the predictive power of the selected set of genes on an independent dataset. We are also working on a multi-modal approach that combines transcriptome data with epigenomic data (and other data possibly) with the final aim of increasing the predictive performances of our model.

## Figures and Tables

**Table 1:** Relevant clinical, pathological and technical metadata of the cohort divided by disease status.

Variable	PD	HC
Gender (male %)	252/390 (64%)	123/189 (65%)
Age at enrollment	62 ± 10	61 ± 11
Disease duration	2 ± 2	-
RBD	37%	20%
TD	70%	13%
Number of sites	25	23
MoCA ≤26 (CI-adjusted for education)	33%	0.5%
RIN	8 ± 1.7	8 ± 1.7

**Table 2:** Average performances of XGBoost over 20 runs of 10-fold cross validation.

	Mean	Standard Deviation
AUC	71.3	1.2
Accuracy	69.3	1.2
Sensitivity	81.7	1.6
Specificity	45.5	2.3
Balanced Accuracy	63.6	1.3
F1	77.8	0.9

**Table 3:** List of the 20 most important protein coding genes and lincRNAs, ordered by importance. LincRNAs are marked with an asterisk. For each gene, four attributes are listed: (i) Up-arrow/Down arrow: significant over/under-expression in PD subjects compared to HC; (ii) HGCN HUGO Gene Nomenclature Committee symbol (or Ensembl ID when missing); (iii) Average XGBoost importance over 20 runs of 5-fold cross validation; (iv) Number of times that a gene is selected over 20 runs of feature selection. For a more complete list, including the genes that are selected 70% of the times, see Table A1.

e	Symbol	imp	f	e	Symbol	imp	f
↑	MYOM1	82.1	20	↑	SLC25A20	62.7	20
	NRM	46.4	20	↓	PHF7	45.9	20
↑	ENSG00000277763 *	39.4	20		ICA1	36	20
↑	CPT1A	33.8	20		LINC02422 *	33.3	20
	GSTM1	32.4	20		PCDHGA6	31.6	20
	AK5	31.5	20	↓	GCNT2	29.9	20
	CERS4	29.7	20	↓	YJU2	29.4	20
	SURF6	27.7	20		ENSG00000281181 *	26.7	20
	ENSG00000285774 *	26.7	20	↑	ENSG00000272688 *	26.2	20
	SERF1B	25.8	20		ENSG00000284773	25.7	20

LincRNAs are marked with an asterisk.

## Data Availability

The pseudocode used for the analysis is reported in Table A1. R codes used to perform the preprocessing and the analysis are available upon request.

## Appendix A

**Algorithm A1** Pseudocode.
1: Let *F* be the total number of features 2: **for***r* = 1 to 20 **do** 3: Divide data into 10 stratified folds using random seed *r* 4: **for** fold *k* = 1 to 10 **do** 5: Set fold *k* as validation_set and the remaining 9 folds as training_set 6: **for** *s* = 1 to 100 **do** 7: Divide training_set into 5 stratified folds using random seed *s* 8: Take 4 of the folds as the new training set 9: Train a RF on this training set with 1000 trees 10: **for** *f* = 1 to *F* **do** 11: Set $is\_outlier_{r,s,f}$ = 0 12: Estimate $importance_{r,s,f}$ 13: **end for** 14: Evaluate $th_{r,s}$ = MEDIAN${}^{\left(f\right)}$($importance_{r,s,f}$) + 1.5 ∗ IQR${}^{\left(f\right)}$($importance_{r,s,f}$) where MEDIAN${}^{\left(f\right)}$ means median over the *F* values of *f* 15: **for** *f* = 1 to *F* **do** 16: $is\_outlier_{r,s,f}$ = IFELSE($importance_{r,s,f}$ > $th_{r,s}$, 1, 0) 17: **end for** 18: **end for** 19: **for** *f* = 1 to *F* **do** 20: Set $percentage\_outlier_{r,f}$ = 0 21: **for** *s* = 1 to 100 **do** 22: $percentage\_outlier_{r,f}$ += $is\_outlier_{r,s,f}$ 23: **end for** 24: **end for** 25: **for** *C* = 1 to 100 **do** 26: Evaluate $is\_selected_{r,f,C}$ = IFELSE($percentage\_outlier_{r,f}$ > *C*, 1, 0) 27: Train XGBoost on the training_set using only features *f* with $is\_selected_{r,f,C}$=1 28: Estimate performance ROCAUC${}_{r,k,C}$ on the validation_set 29: **end for** 30: **end for** 31: **end for** 32: **for***C* = 1 to 100 **do** 33: Evaluate m_ROCAUC${}_{r,C}$ = MEDIAN${}^{\left(k\right)}$(ROCAUC${}_{r,k,C}$) over the 10 values of *k* 34: **end for** 35: **for***C* = 1 to 100 **do** 36: Evaluate m_ROCAUC${}_{C}$ = MEDIAN${}^{\left(r\right)}$(ROCAUC${}_{r,C}$) over the 20 values of *r* 37: **end for** 38: Let $C^{*}$ = ARGMAX${}_{C}$(m_ROCAUC${}_{C}$) 39: **for***f* = 1 to *F* **do** 40: Set $count\_selected_{f}$ = 0 41: **for** *r* = 1 to 20 **do** 42: $count\_selected_{f}$ += $is\_selected_{r,f,C^{*}}$ 43: **end for** 44: **end for**

**Table A1:** Complete list of the most frequent protein coding genes and lincRNAs, ordered by importance. LincRNAs are marked with an asterisk. For each gene, four attributes are listed: (i) No arrow, an upward pointing arrow, a downward pointing arrow indicate no significant DE bewteen PD and HC, significant over-expression in PD subjects, significant under-expression in PD subjects, respectively; (ii) HGCN symbol (or Ensembl ID when missing); (iii) Average importance over 20 runs of 5-fold cross validation; (iv) Frequency of occurrence over 20 repetitions.

e	Symbol	imp	f	e	Symbol	imp	f	e	Symbol	imp	f
↑	MYOM1	82.1	20	↑	SLC25A20	62.7	20		NRM	46.4	20
↓	PHF7	45.9	20	↑	ENSG00000277763 *	39.4	20		ICA1	36	20
↑	CPT1A	33.8	20		LINC02422 *	33.3	20		GSTM1	32.4	20
	PCDHGA6	31.6	20		AK5	31.5	20	↓	GCNT2	29.9	20
	CERS4	29.7	20	↓	YJU2	29.4	20		SURF6	27.7	20
	ENSG00000281181 *	26.7	20		ENSG00000285774 *	26.7	20	↑	ENSG00000272688 *	26.2	20
	SERF1B	25.8	20		ENSG00000284773	25.7	20		TREML4	25.7	20
	ANKRD34B	25.1	14	↓	NDUFB9	25	20	↓	ERLIN2	24.6	20
	ENSG00000276651 *	24.4	20		CCR4	23.9	20		NFE2L3	23.9	20
	FGGY	23.5	20		ENSG00000234902 *	23.3	20	↑	ARRDC4	23.1	20
	TMTC4	21.6	20	↓	BPHL	20.1	20		C2orf42	20	20
	BTBD19	19.8	15		LOXHD1	19.4	20		DHFR	19.1	20
	LINC02470 *	18.9	20		SHISA4	18.8	20	↑	FKBP5	18.4	20
	ENSG00000234426 *	18.3	20	↑	TKTL1	18.1	20		ATP6V0A2	18	20
	GPR19	17.9	20		ZNF584	17.4	20		FAN1	17.4	20
	MRPS6	17.2	18		TSPAN2	16.9	20	↓	CRAT	16.5	20
	CCRL2	16	20		GTF2IRD2	15.9	20		PUDP	15.8	20
↓	NOP16	15.7	20	↑	LINC00243 *	15.6	20	↑	CEP19	15.6	20
	GAB3	15.6	20	↓	ENSG00000269399 *	15.5	20	↓	YOD1	15.3	20
	GET1	15.3	19	↓	NREP	15.2	20		YES1	14.8	15
↑	COL9A3	14.5	20		NSUN4	14.4	20	↓	FARSB	14.3	20
↑	GZMB	14.2	20		B4GALNT3	14.1	20	↓	TBL2	14.1	20
↓	RAP1GAP	13.7	20	↑	BASP1	13.5	20		PRUNE2	13.5	19
	FBN2	13.3	20		VNN1	13.2	20	↑	LSMEM1	13.2	20
	ZSCAN21	13.1	20		CLEC12A	13	20	↓	COA4	13	20
↓	DPY19L2	12.9	20	↑	RNASET2	12.8	20		DCXR	12.4	20
	WDR49	12.4	20		CRYZ	12.3	15	↑	LINC00623 *	12.2	20
↓	ZNF714	12.2	20	↑	TOR1B	12	20	↑	ADGRE5	11.9	20
↑	SULF2	11.7	20		MSH3	11.7	20		PCDHGB3	11.6	20
	SPHK1	11.3	20	↓	G6PC3	11	20	↓	MASTL	11	20
↓	LINC01806 *	11	20	↓	SQLE	11	20		PWP2	10.9	20
	TXLNB	10.8	20		ZSWIM3	10.7	19		SFXN4	10.6	20
↑	RUBCNL	10.6	20	↓	PNO1	10.2	20		SMIM12	10.2	18
	TNFRSF10B	10.2	20		GPR162	10	16	↓	KRT1	10	20
	B3GAT1	9.8	20		PILRB	9.8	20	↑	TAP2	9.8	20
	MSR1	9.7	18		LINC00482 *	9.6	18		OSER1-DT *	9.4	15
	ASCC1	9.4	20	↑	ZNF429	9.4	20		SSPN	9.3	20
↓	GYPA	9.3	20	↑	FAT4	9.3	20	↓	SLC18B1	9.2	20
	TIPIN	9.1	20		IL18RAP	9	15		GYPE	8.9	20
↓	HYAL3	8.7	20	↑	PREX1	8.7	19		KLRC2	8.6	20
	FCER1A	8.6	19		ENDOG	8.6	20		GSTM3	8.6	20
↑	TMEM252	8.6	20		SRGAP2C	8.5	17	↓	ATP5MC1	8.5	20
	FIS1	8.3	20		ARRDC3-AS1 *	8.3	19		LINC01948 *	8.3	16
	TPPP3	8.2	20		HDHD3	8.2	19	↓	LINC01560 *	8.2	20
↓	IFRD2	8.1	20	↓	STK11	8.1	17	↓	TARBP1	7.9	20
	LINC00299 *	7.9	19		XCL1	7.8	20		ZNF491	7.6	20
	LINC00570 *	7.4	20		PARS2	7.4	20		INPP1	7.4	20
↓	TMEM245	7.4	20		NECAP2	7.3	19		PER3	7.3	20
	CDCA4	7.2	20		NUP210L	7.2	19		GTF2H2	7.1	20
	APOLD1	7.1	20		ETFDH	7	20	↓	GPX4	6.9	20
↓	PPP1R14B	6.9	20	↓	GOLGA6L9	6.9	20		ATP6AP1L	6.9	20
↓	GFUS	6.9	20	↑	ENSG00000268240 *	6.7	20		MAST4	6.7	20
↑	ENSG00000225750 *	6.7	20	↑	BCL6	6.5	20		DDO	6.5	20
↑	TMEM185B	6.4	20		UPB1	6.4	20		CCR3	6.4	20
	PLIN2	6.4	20	↓	RALY-AS1 *	6.3	20		DDIT4	6.3	20
	FGFR2	6.3	20	↓	PAICS	6.3	20		ENSG00000278384	6.2	19
	HLA-DRB5	6.2	16	↑	VDR	6.1	19		ENSG00000254789 *	6.1	20
	ENSG00000260077 *	6.1	18		CLEC18A	6.1	15	↑	LINC02193 *	6.1	20
↑	SBNO1	6.1	20	↑	VAV1	6.1	17		SCN3A	6	20
	CCL4L2	5.9	20		ASB3	5.8	18		GSTM2	5.8	20
↑	KDM5B	5.8	20		GNAL	5.7	18		KCNMB1	5.6	15
	CSGALNACT1	5.6	20	↓	RNASEH1	5.6	20		ENSG00000285476	5.5	20
↑	FBXL13	5.4	20		VLDLR	5.4	20	↑	FPR2	5.4	20
↑	PPP1R3B	5.4	20	↓	SRSF8	5.4	20		APOO	5.3	20
↑	TXNIP	5.3	20	↓	MPG	5.3	19		TAS2R43	5.2	20
	FLVCR2	5.1	20		SLC25A3	5.1	20	↓	CD36	5.1	19
	CENPK	5	18		C5	5	14	↑	PRRG4	5	20
	DYRK1B	4.9	17	↑	APTR *	4.8	20	↓	TMEM14C	4.8	19
	PF4V1	4.8	20	↓	ZNF789	4.7	20		UBR7	4.7	17
	HMOX2	4.6	19		PID1	4.6	20		LERFS *	4.5	20
	ENSG00000266302	4.5	20		AKAP5	4.5	20		DPCD	4.4	20
	TMTC2	4.4	20		NKAP	4.4	17		ENSG00000276476 *	4.4	20
	EDAR	4.4	20		VSTM1	4.4	20		PDK4	4.3	20
↑	HIF1A	4.3	20	↓	GRHPR	4.3	20		TUBB2A	4.3	20
	PALLD	4.3	20	↑	LINC01303 *	4.3	20		FPR3	4.3	20
↑	TMEM45B	4.3	20		RGMB	4.3	20	↑	CREM	4.3	20
↓	LYRM9	4.3	18		VSIG10	4.3	20	↓	TSPAN17	4.2	20
	BBLN	4.1	16		LTA4H	4.1	20		U2AF1	4.1	20
	PPAN	4.1	20		ARL17B	4.1	20		ENSG00000274922 *	4.1	19
	TM9SF1	4	20		EPPK1	4	20	↑	THBD	4	20
	DRAXIN	4	14	↓	USP12	3.9	20		SLC11A2	3.9	19
	ENSG00000259071 *	3.9	20	↑	SPON2	3.8	20		ENSG00000256427 *	3.8	14
	FAM124B	3.8	20	↓	NBDY	3.8	20	↓	MBNL3	3.8	20
↓	COMMD9	3.7	20		CTSK	3.7	20	↓	CYREN	3.7	20
↑	LINC00654 *	3.7	20	↑	ENSG00000270972 *	3.6	20	↓	SVBP	3.6	20
	TMEM185A	3.6	18	↓	CDK6	3.6	20		MFSD9	3.6	20
	CRTAP	3.5	14		CSTB	3.5	20	↓	PTRHD1	3.5	20
	PPIE	3.5	20		HLA-DMB	3.5	15		DSC1	3.5	20
↓	CEP85	3.4	20		RNF182	3.4	20	↓	HSD17B8	3.4	20
	NKX3-1	3.4	20		F2R	3.4	20		ENSG00000224635 *	3.4	19
↓	HDHD5	3.4	20		ZKSCAN4	3.4	20	↑	KPNB1	3.3	20
↑	LAMP1	3.3	20	↓	ENSG00000277369 *	3.3	20	↓	SNHG4 *	3.3	20
	MYG1	3.3	15	↓	SLC25A25	3.3	18		U2AF1L5	3.3	20
↓	ETHE1	3.2	20		KAT2B	3.2	20	↓	MIR378D2HG *	3.2	16
↓	TLCD4	3.2	20	↑	SPTY2D1	3.2	20		MYOM2	3.2	20
↑	IL18R1	3.1	20	↓	UBE2E2	3.1	20	↑	KREMEN1	3.1	20
	ENSG00000227920 *	3.1	19		COX5A	3	16	↓	LINC00920 *	3	20
	NRG1	3	17	↓	GPR15	3	20	↓	UROS	3	20
	LINC02520 *	3	20	↑	TGM3	3	20		CCZ1B	3	20
	S100B	3	20		NR4A2	2.9	20		SULT1A1	2.9	19
	TMEM273	2.9	20		LINC00381 *	2.9	18		FMN1	2.8	20
	CCDC144A	2.8	20	↑	LMTK2	2.8	20	↑	HSDL2	2.8	20
↑	BMX	2.8	20		ZNF559	2.8	20	↑	ELL	2.8	17
↑	MIR646HG *	2.8	20	↓	CREG1	2.8	20		DACT1	2.8	19
↑	TBC1D30	2.7	20		JUN	2.7	20		CLEC4F	2.7	20
	ENSG00000259652 *	2.7	19	↓	POMC	2.6	14	↓	THAP7	2.6	20
	YDJC	2.6	20	↑	NFE4	2.6	20		PDZD4	2.6	20
	FTCDNL1	2.6	20	↑	GABARAPL1	2.6	20	↓	TIMM9	2.6	20
↓	ANKRD9	2.6	19	↓	RNF11	2.5	19		ATP6V1F	2.5	20
↓	MTCH2	2.5	20		SCO1	2.5	19		NOTCH2NLA	2.5	20
	GATD3A	2.5	20		MAP3K7CL	2.5	20	↑	NCAM1	2.4	20
	LINC02273 *	2.4	20		PI16	2.4	14	↑	CLCN4	2.4	20
	CTXN2-AS1 *	2.4	19	↓	MECR	2.4	20	↑	ENSG00000273243 *	2.4	20
	COL18A1	2.4	20	↑	TLK2	2.3	20	↓	HMBS	2.3	17
	CCDC102A	2.3	15		TTF2	2.3	19	↓	C16orf91	2.3	16
↑	HERPUD1	2.3	20	↑	SLA	2.2	20		TMEM102	2.2	20
	HLA-DQB1	2.2	20		DUSP19	2.2	20	↓	KCTD3	2.2	14
	FOLR3	2.2	20		C1orf220 *	2.1	15	↑	PRDM8	2.1	19
↑	KIF1B	2.1	19		LINC00298 *	2.1	18		LINC01410 *	2.1	20
↑	LINC02218	2.1	20	↑	NKAPL	2.1	20		RAB34	2.1	20
↓	GSTZ1	2.1	19	↓	ENSG00000267575 *	2.1	16		SYNM	2	17
↑	RNF149	2	20	↑	CSRNP1	2	17		LSG1	2	19
↑	TOP1	2	20	↑	IRF1	2	14	↑	SYTL3	2	20
	ZNRD2	2	20	↓	ICAM4	2	20		CLEC12B	1.9	20
↑	NDRG3	1.9	20		PAQR8	1.9	20	↓	LGALS2	1.9	20
↓	WDR11	1.9	17	↓	HDAC9	1.9	20		RRS1	1.9	15
	ANKRD55	1.9	16	↓	NIT2	1.8	14		ENSG00000272908 *	1.8	20
↓	PMVK	1.8	20		RFC5	1.8	20	↓	PRADC1	1.8	18
↑	HSD17B13	1.8	18	↑	ZNF487	1.8	20	↑	NUP50-DT *	1.8	20
	TOR3A	1.7	20		ADAM15	1.7	20		ENSG00000285492 *	1.7	20
↑	CA4	1.7	20		PARN	1.7	18	↓	AKR1A1	1.7	20
↑	DOCK4	1.7	20	↑	IRS2	1.7	20		CHST2	1.7	20
↓	C3orf18	1.6	20		ZNF69	1.6	20	↑	CCN3	1.6	20
	CLMN	1.6	20		GCAT	1.6	14	↓	TXN2	1.6	15
↑	TPST1	1.6	20	↑	MIR3945HG *	1.5	20		PTPRN2	1.5	20
	ADGRB3	1.5	18		ENSG00000281831 *	1.5	15	↓	EIF2D	1.5	20
	OAS1	1.5	14	↑	ACSL1	1.5	20		SRP19	1.5	20
↑	NUP50	1.5	20	↓	XK	1.5	20	↑	COA1	1.4	19
	KRT72	1.4	20	↑	ROPN1L	1.4	16		SLC25A43	1.4	20
↑	ENSG00000251093 *	1.4	20	↑	ABCA1	1.4	19		AFDN	1.4	18
	TMEM176B	1.3	20	↑	SERINC3	1.3	18	↑	CEMIP2	1.3	20
↓	NAXD	1.3	20		NFXL1	1.3	20	↓	ALKBH7	1.3	19
	ENSG00000259959 *	1.3	20	↓	ENSG00000275765 *	1.3	15		BSCL2	1.2	18
↓	CISD2	1.2	20	↑	DCAF4L1	1.2	19	↑	CD93	1.2	19
↓	APRT	1.2	20		CYBRD1	1.1	16	↑	NBPF26	1.1	20
↓	MRPS27	1.1	18		GIMAP1	1.1	20		RRP7A	1.1	20
	ISCA1	1.1	20		FADS2	1.1	19	↑	TRANK1	1.1	18
↑	PHACTR1	1.1	20	↑	VNN3	1	20	↑	HLX	1	20
↑	JADE1	1	20	↓	KNOP1	1	20		HLA-DQA2	1	19
	XKR3	1	20		P2RX4	0.9	16		CPA3	0.9	19
↓	C8orf33	0.9	19		MS4A4E	0.9	20		ENSG00000274979 *	0.9	20
↑	RPGRIP1	0.9	14	↑	NCR1	0.9	20	↑	PRF1	0.9	20
	PEA15	0.8	19		S100A10	0.8	19		ERO1A	0.8	20
↑	ADGRG3	0.8	16	↑	BTNL8	0.8	20		EMC9	0.8	20
	LONRF3	0.8	20		SLC38A11	0.7	20	↑	BAZ1A	0.7	17
↓	ACAD11	0.7	15	↓	C1orf109	0.7	20		SUV39H1	0.7	14
	PAAF1	0.7	18		MGST3	0.7	20	↑	PHTF1	0.7	20
↑	CD55	0.6	20		MTPAP	0.6	20		ZNF80	0.6	18
↑	SIPA1L2	0.6	20		PTGDS	0.6	19	↓	SNX3	0.6	20
	KLF9	0.6	17	↑	TGFA	0.6	20		HLA-DQA1	0.5	20
	AMACR	0.5	20		NCAPG2	0.5	14	↓	CTSH	0.5	15
↑	ENSG00000282988	0.5	17		PANX1	0.5	20		HLA-A	0.5	20
↑	CPD	0.5	20	↑	NHS	0.4	16		KRT73	0.4	20
↑	METRNL	0.3	17	↓	PIGW	0.3	16	↑	AVIL	0.3	20
↑	ABCG1	0.2	20	↑	RAB27A	0.2	20	↑	DNAJC3	0	20

LincRNAs are marked with an asterisk.
